# Association between mean airway pressure during high-frequency oscillatory ventilation and pulmonary air leak in extremely preterm infants during the first week of life

**DOI:** 10.3389/fped.2024.1410627

**Published:** 2024-05-30

**Authors:** Kei Tamai, Akihito Takeuchi, Makoto Nakamura, Kazue Nakamura, Naomi Matsumoto, Takashi Yorifuji, Misao Kageyama

**Affiliations:** ^1^ Division of Neonatology, NHO Okayama Medical Center, Okayama, Japan; ^2^ Department of Epidemiology, Okayama University Graduate School of Medicine, Dentistry and Pharmaceutical Sciences, Okayama, Japan

**Keywords:** high-frequency oscillatory ventilation, mean airway pressure, pneumothorax, preterm infant, pulmonary air leak

## Abstract

**Background:**

While positive pressure ventilation has been considered an important contributing factor associated with pulmonary air leaks, studies examining the association between specific ventilatory settings during acute-phase high-frequency oscillatory ventilation (HFOV) and pulmonary air leaks among extremely preterm infants are limited.

**Methods:**

This was a single-center retrospective cohort study conducted at an institution that primarily used HFOV after intubation in extremely preterm infants. We analyzed data from extremely preterm infants born between 2010 and 2021. The primary outcome was pulmonary air leakage during the first 7 days of life. The exposure variable was the maximum mean airway pressure (MAP) on HFOV during the first 7 days of life or before the onset of pulmonary air leaks. Maximum MAP was categorized into three groups: low (7–10 cmH_2_O), moderate (11–12 cmH_2_O), and high (13–15 cmH_2_O) MAP categories. We conducted robust Poisson regression analyses after adjustment for perinatal confounders, using the low MAP category as the reference.

**Results:**

The cohort included 171 infants (low MAP, 123; moderate MAP, 27; and high MAP, 21). The median (interquartile range) gestational age and birth weight were 25.7 (24.3–26.7), 25.7 (24.9–26.9), and 25.3 (24.3–26.6) weeks and 760 (612–878), 756 (648–962), and 734 (578–922) g for infants in the low, moderate, and high MAP categories, respectively. Compared to infants in the low MAP category, those in the high MAP category had a higher incidence of pulmonary air leaks (4.1% vs. 33.3%; adjusted risk ratio, 5.4; 95% confidence interval, 1.6–18.5). In contrast, there was no clear difference in the risk of pulmonary air leaks between the moderate and low MAP categories (3.7% vs. 4.1%; adjusted risk ratio, 0.9; 95% confidence interval, 0.1–6.1).

**Conclusion:**

Extremely preterm infants requiring high MAP (≥13 cmH_2_O) in acute-phase HFOV had a higher risk of pulmonary air leak during the first 7 days of life.

## Introduction

Pulmonary air leak is a serious complication in the neonatal intensive care unit (NICU) and is associated with mortality (1–3), bronchopulmonary dysplasia (1), and intraventricular hemorrhage (4, 5). Among preterm and term neonates, pulmonary air leak typically occurs within the first 7 days of life (6, 7). This period is characterized by significant vulnerability and instability, particularly in extremely preterm infants. Several risk factors for pulmonary air leak have been identified, including prematurity, positive pressure ventilation, and respiratory distress syndrome (8). While positive pressure ventilation has been considered an important contributing factor associated with pulmonary air leaks (9), studies examining the association between specific ventilatory settings and pulmonary air leaks among extremely preterm infants are limited. A single-center retrospective cohort study conducted in Brazil suggested that pressure-related ventilatory settings during intermittent mandatory ventilation (IMV) in extremely low birth weight infants were not associated with a higher risk of pneumothorax in the first 7 days of life (10). However, to date, no studies have evaluated the association between ventilatory settings on acute-phase high-frequency oscillatory ventilation (HFOV) and pulmonary air leak in extremely preterm infants.

Therefore, this study aimed to investigate the association between the maximum mean airway pressure (MAP) on acute-phase HFOV and pulmonary air leaks during the first 7 days of life in extremely preterm infants. Understanding the ventilatory parameters of acute-phase HFOV associated with a higher risk of pulmonary air leak could help clinicians provide more careful ventilation with adequate oxygenation while avoiding the risk of inducing pulmonary air leak.

## Materials and methods

### Study cohort

This single-center retrospective cohort study was conducted to analyze the perinatal data of extremely preterm infants born between January 2010 and December 2021 who were admitted to our tertiary NICU. Infants with out-of-hospital births, major congenital anomalies, or pulmonary air leaks in the delivery room were excluded. As this study focused on the effects of initial and acute HFOV support on the occurrence of pulmonary air leaks, we also excluded infants who experienced pulmonary air leaks after the first 7 days of life, during IMV, or those who did not require invasive respiratory management during the first 7 days of life. All perinatal data, including variables other than potential confounders, were obtained from the discharge summary and verified using admission charts.

Extremely preterm infants were intubated when they exhibited poor spontaneous respiration, respiratory distress, and/or low oxygen saturation on continuous positive airway pressure. Subsequently, respiratory management was initiated with HFOV, primarily using Humming V or Humming Vue ventilators (Metran Co., Ltd., Saitama, Japan). Physicians adjusted the ventilator settings based on respiratory patterns, oxygen saturation, and blood gas results. Our unit's routine HFOV settings are Frequency set to 15 Hz. The target oxygen saturation and CO_2_ range for extremely preterm infants during the first 7 days of life in our NICU were set at 90%–95% and 40–50 mmHg, respectively. We rarely switched from HFOV to IMV because of hypotension or a deteriorating circulatory or respiratory status. We lowered MAP when radiographic findings suggesting pulmonary hyperinflation, such as flattening of the diaphragm, were identified.

### Maximum MAP on acute-phase HFOV

The maximum MAP for acute-phase HFOV was defined as the highest MAP for HFOV before the onset of pulmonary air leakage or within the first 7 days of life for infants without pulmonary air leakage. The maximum MAP values were categorized into three groups based on the MAP values of the inclusion criteria used in previous randomized controlled trials (low- and high-volume strategies) comparing HFOV with conventional ventilation: low (7–10 cmH2O), moderate (11–12 cmH2O), and high (13–15 cmH2O) MAP categories (11, 12).

### Pulmonary air leak

Pulmonary air leaks include pneumothorax, pulmonary interstitial emphysema, pneumomediastinum, and pneumopericardium. The diagnosis of pulmonary air leak in this study was determined based on medical records and discharge summaries, and the authors also reviewed the radiographs to confirm that the diagnosis of pulmonary air leak was correct.

### Statistical analyses

We first compared the baseline characteristics of extremely preterm infants according to the maximum MAP categories: low (7–10 cmH2O), moderate (11–12 cmH2O), and high (13–15 cmH2O). The differences between the groups in the low, moderate, and high MAP categories were tested using the *χ*^2^ test or Kruskal–Wallis test. We also evaluated the respiratory characteristics of extremely preterm infants with pulmonary air leaks. Finally, we conducted Poisson regression models with robust variance estimators to investigate the associations between the MAP categories and the occurrence of pulmonary air leaks. We estimated the risk ratios (RRs) and 95% confidence intervals (CIs) for the main outcomes using the low MAP category as a reference after adjusting for potential confounders. We selected possible potential confounders based on previous studies (1, 10, 13), including gestational age (22–23, 24–25, and 26–27 gestational weeks, categorical), pulmonary surfactant administration (dichotomous), prolonged preterm premature rupture of membranes (dichotomous), and clinical risk index for babies (CRIB) II score (≤9, 10–14, and ≥15, categorical) (14). In further analyses, we excluded infants who received IMV before the occurrence of pulmonary air leak or within the first 7 days of life. This exclusion was implemented to ensure a more precise examination of the association between the MAP in acute-phase HFOV and the occurrence of pulmonary air leaks. A sensitivity analysis was conducted, incorporating the HFOV rate as a continuous confounding variable. The HFOV rate was defined as the rate of time spent on HFOV before the onset of pulmonary air leak in cases with this complication. For cases without pulmonary air leak, it was defined as the ratio of time spent on HFOV until 7 days of age, equivalent to 168 h, or until death before 7 days of age.

Gestational age was determined based on an ultrasound examination during the first trimester and date of the last menstrual period. The diagnosis of respiratory distress syndrome was based on clinical and radiographic findings and a stable microbubble test of gastric aspirates (15). When the diagnosis of respiratory distress syndrome was made, we intubated and administered a pulmonary surfactant at 120 mg/kg (beractant). Pulmonary surfactant administration was defined as surfactant administration during the first 7 days of life. Premature rupture of membranes was defined as the rupture of the amniotic sac before labor began. Prolonged preterm premature rupture of membranes was defined as birth more than 7 days after the onset of premature rupture of membranes. The CRIB II score was calculated using the following five variables measured upon admission to the NICU: sex, gestational age, birth weight, base excess, and body temperature (14). Base excess was determined from the first blood gas analysis through a retrospective chart review. *P* values less than 0.05 were considered significant. Stata SE version 16 statistical software (Stata Corp., College Station, TX, US) was used for all analyses.

This study was approved by the Internal Review Board of Okayama Medical Center (RINKEN 2023-005) and was conducted according to the principles of the Declaration of Helsinki. Informed consent was waived because of the retrospective nature of the study.

## Results

**Figure 1:**
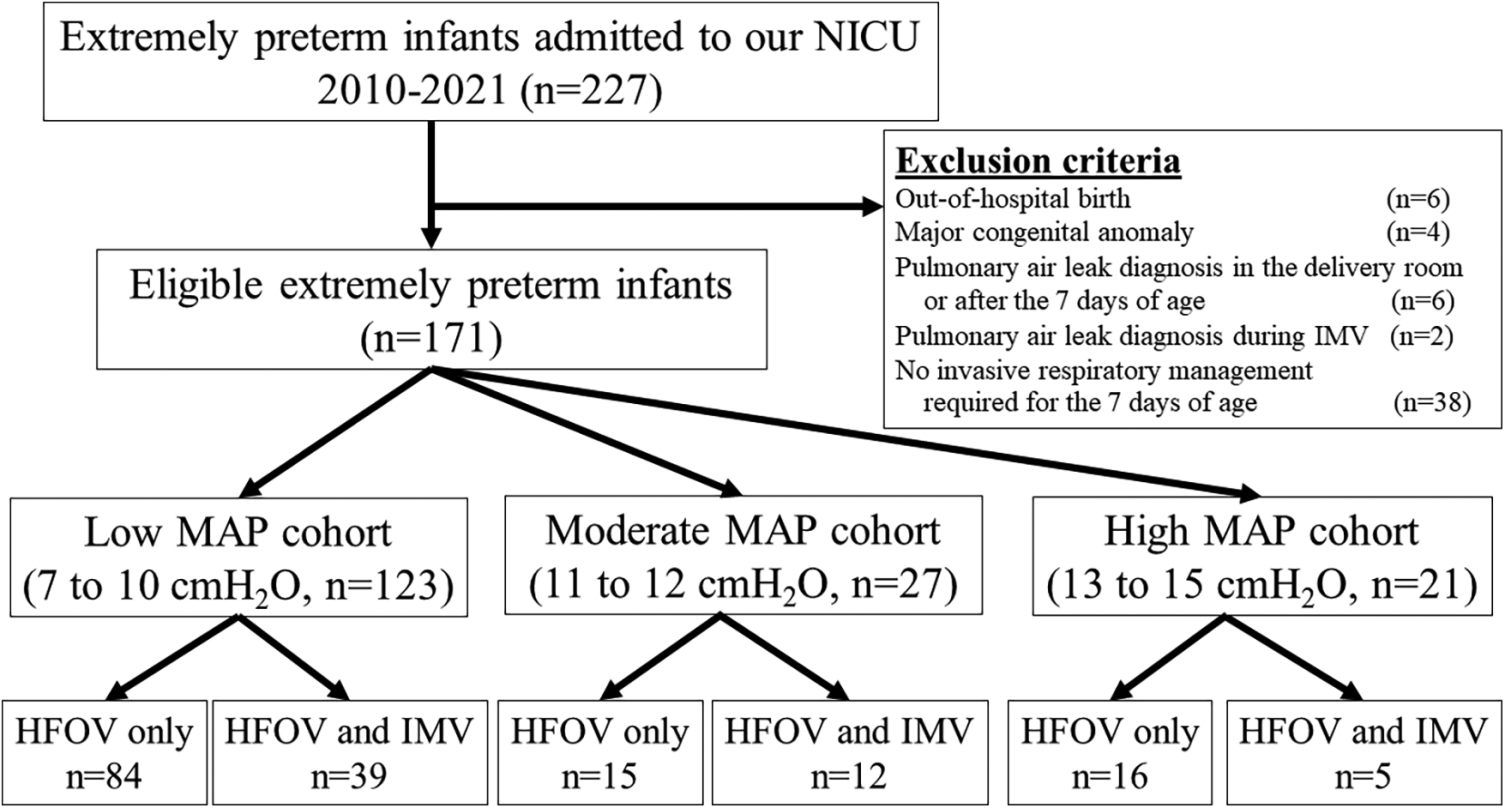
Study flow chart. HFOV, high-frequency oscillatory ventilation; IMV, intermittent mandatory ventilation; MAP, mean airway pressure; NICU, neonatal intensive care unit.

Between 2010 and 2021, 227 extremely preterm infants were admitted to the NICU. Of these infants, 56 were excluded: out-of-hospital birth (*n* = 6), congenital anomalies (*n* = 4), pulmonary air leak diagnosis in the delivery room or after the first 7 days of life (*n* = 6), pulmonary air leak diagnosis during IMV (*n* = 2), and no invasive respiratory management during the first 7 days of life (*n* = 38). Finally, 171 extremely preterm infants were included in the study (Figure 1). In the low, moderate, and high MAP cohorts, 84, 15, and 16 infants, respectively, received HFOV exclusively during the first 7 days of life. None of the infants in our study cohort exclusively received IMV.

Table 1 presents the characteristics of eligible infants categorized according to MAP categories. Infants in the high MAP category were likely to have a higher incidence of prolonged premature rupture of membranes, surfactant administration, and persistent pulmonary hypertension of the newborn. Moreover, the infants in the high MAP category tended to have a high fraction of inspired oxygen and a high rate of intraventricular hemorrhage.

**Table 1:** Characteristics of eligible extremely preterm infants by maximum MAP category (*n* = 171).

	Maximum MAP category			
	Low MAP 7–10 cmH_2_O	Moderate MAP 11–12 cmH_2_O	High MAP 13–15 cmH_2_O	*P*-value^a^
	*n* = 123	*n* = 27	*n* = 21	
Gestational age, weeks	25.7 (24.3–26.7)	25.7 (24.9–26.9)	25.3 (24.3–26.6)	0.67
Birth weight, g	760 (612–878)	756 (648–962)	734 (578–922)	0.69
Sex, male	73 (59.3)	17 (63.0)	11 (52.4)	0.76
CRIB II score	11.5 (10–13)	12 (10–14)	12 (11–16)	0.22
Antenatal corticosteroids	108 (87.8)	24 (88.9)	18 (85.7)	0.95
Cesarean section	67 (54.5)	13 (48.1)	8 (38.1)	0.36
Premature rupture of membrane	40 (32.5)	9 (33.3)	12 (57.1)	0.09
Prolonged premature rupture of membrane, ≥7 days	11 (8.9)	4 (14.8)	6 (28.6)	0.04
Administration of surfactant	95 (77.2)	25 (92.6)	20 (95.2)	0.04
Respiratory distress syndrome	81 (65.9)	16 (59.3)	9 (42.9)	0.13
Persistent pulmonary hypertension of the newborn	15 (12.2)	9 (33.3)	17 (81.0)	<0.01
Maximum fraction of inspired oxygen				<0.01
21%–39%	74 (60.2)	3 (11.1)	1 (4.8)	
40%–69%	30 (24.4)	9 (33.3)	3 (14.3)	
70%–100%	19 (15.4)	15 (55.6)	17 (81.0)	
HFOV rate, %^b^	100 (69–100)	97 (55–100)	100 (91–100)	0.40
Mortality	10 (8.1)	4 (14.8)	3 (14.3)	0.45
Intraventricular hemorrhage, grade 1–4	45 (36.6)	12 (44.4)	15 (71.4)	0.01
Severe intraventricular hemorrhage, grade 3–4	8 (6.5)	3 (11.1)	5 (23.8)	0.04

Data are expressed as *n* (%) or median (interquartile range).

CRIB, clinical risk index for babies; HFOV, high-frequency oscillatory ventilation; MAP, mean airway pressure.

^a^ The differences between the three MAP categories were tested using the *χ*^2^ test or Kruskal-Wallis test.

^b^ The HFOV rate was defined as the rate of time spent on HFOV until the onset of pulmonary air leak, 7 days of age, or death before 7 days of age.

The respiratory data of extremely preterm infants with pulmonary air leaks are listed in Table 2. The study included cases of pneumothorax and pulmonary interstitial emphysema; however, no cases of pneumomediastinum or pneumopericardium were identified. Pneumothorax alone was the most common type of pulmonary air leak (61.5%).

**Table 2:** Respiratory data in extremely preterm infants with pulmonary air leaks.

	Pulmonary air leaks	
	(*n* = 13)	
Age at pulmonary air leaks, hours	50	(33–115)
Bilateral pulmonary air leaks	3	(23.1)
Chest tube for pneumothorax	10/11	(90.9)
Types of pulmonary air leaks		
Pneumothorax only	8	(61.5)
Pulmonary interstitial emphysema only	2	(15.4)
Pneumothorax and pulmonary interstitial emphysema	3	(23.1)
Pneumomediastinum	0	(0)
Pneumopericardium	0	(0)
Ventilator settings at pulmonary air leaks		
MAP, cmH_2_O	10	(9–12)
Fraction of inspired oxygen, %	30	(22–60)

Data are expressed as *n* (%), *n*/*N* (%), or median (interquartile range).

MAP, mean airway pressure.

Table 3 lists the associations between MAP categories and pulmonary air leaks. After adjustment for potential confounders, infants in the high MAP category had a higher incidence of pulmonary air leak than those in the low MAP category (33.3% vs. 4.1%, adjusted RR 5.4, 95% CI 1.6–18.5). There was no clear difference in the risk of pulmonary air leaks between the moderate and low MAP categories (3.7% vs. 4.1%, adjusted RR 0.9, 95% CI 0.1–6.1).

**Table 3:** Associations between maximum MAP on acute-phase HFOV and pulmonary air leak among eligible extremely preterm infants (*n* = 171).

	Pulmonary air leaks/	% of pulmonary air leaks	RR (95% CI)			
	Total number		Crude		Adjusted^a^	
Maximum MAP category						
Low MAP, 7–10 cmH_2_O	5/123	4.1	1	(reference)	1	(reference)
Moderate MAP, 11–12 cmH_2_O	1/27	3.7	0.9	(0.1–7.5)	0.9	(0.1–6.1)
High MAP, 13–15 cmH_2_O	7/21	33.3	8.2	(2.9–23.5)	5.4	(1.6–18.5)

Data are presented as the raw number (%) and RR (95% CI).

CI, confidence interval; CRIB, clinical risk index for babies; HFOV, high-frequency oscillatory ventilation; MAP, mean airway pressure; RR, risk ratio.

^a^ Adjusted for gestational age, pulmonary surfactant administration, prolonged premature rupture of membranes, and CRIB II score.

A further analysis was conducted by excluding 56 infants who received even brief sessions of IMV, focusing solely on those who exclusively received HFOV. Although we could not estimate the RR in the moderate MAP group, similar findings were obtained (Table 4).

**Table 4:** Associations between maximum MAP on acute-phase HFOV and pulmonary air leak, excluding infants who received IMV (*n* = 115).

	Pulmonary air leaks/	% of pulmonary air leaks	RR (95% CI)			
	Total number		Crude		Adjusted^a^	
Maximum MAP category						
Low MAP, 7–10 cmH_2_O	4/84	4.8	1	(reference)	1	(reference)
Moderate MAP, 11–12 cmH_2_O	0/15	0	NE		NE	
High MAP, 13–15 cmH_2_O	7/16	43.8	9.2	(3.0–27.9)	7.6	(1.6–36.4)

Data are presented as the raw number (%) and RR (95% CI).

CI, confidence interval; CRIB, clinical risk index for babies; HFOV, high-frequency oscillatory ventilation; IMV, intermittent mandatory ventilation; MAP, mean airway pressure; NE, not estimable; RR, risk ratio.

^a^ Adjusted for gestational age, pulmonary surfactant administration, prolonged premature rupture of membranes, and CRIB II score.

The Supplementary Table 1 shows the results of the sensitivity analysis. Even with the inclusion of the HFOV rate as a potential confounder, no substantial changes in the results were observed.

## Discussion

In this study, we investigated the association between the maximum MAP on acute-phase HFOV and pulmonary air leaks during the first 7 days of life among extremely preterm infants. We found that extremely preterm infants who needed a high MAP (≥13 cmH2O) on acute-phase HFOV were more likely to have a pulmonary air leak during the first 7 days of life.

A few studies have examined the association between detailed acute-phase ventilatory settings and pulmonary air leaks in extremely preterm infants. A single-center retrospective cohort study conducted in Brazil showed that positive end-expiratory pressures or peak inspiratory pressures in extremely low birth weight infants were not associated with a higher risk of pneumothorax during the first 7 days of life (10). In contrast, several previous studies reported that high peak inspiratory pressures and prolonged inspiratory times were associated with the incidence of pneumothorax in very low birth weight infants (13, 16). A case-control study in Canada also demonstrated an association between MAP levels and pneumothorax occurrence during the first 5 days of age in very low birth weight infants (17). However, these studies were reported from institutions that primarily used synchronized IMV rather than HFOV for acute respiratory management of preterm infants. A single-center case-control study in Spain showed that preterm infants born up to 30 gestational weeks who developed pulmonary interstitial emphysema required a higher MAP 24 h before diagnosis compared to those without pulmonary interstitial emphysema (18). To the best of our knowledge, no studies have evaluated the association between MAP in acute-phase HFOV and pulmonary air leaks among extremely preterm infants.

The potential advantages of HFOV over conventional ventilation include lower peak airway pressures, the ability to adequately and independently manage oxygenation and ventilation while using very small tidal volumes, and preservation of normal lung architecture even at high mean airway pressures (19). Therefore, HFOV is also believed to reduce the risk of pulmonary air leaks (8). However, a systematic review described that the use of HFOV could lead to an increased incidence of pulmonary air leaks compared with conventional ventilation among preterm infants (20). The present study revealed that extremely preterm infants who needed high MAP (≥13 cmH2O) on acute-phase HFOV had a higher risk of pulmonary air leaks than those who did not require a high MAP. This finding suggests that excessive pressure and volume in premature lungs are the causes of pulmonary air leaks during acute-phase HFOV, which is generally consistent with the results of studies using conventional ventilation (13, 16, 17). In contrast, it should be recognized that pulmonary hypertension, resulting in desaturation requiring high oxygen concentrations, may have contributed to increased MAP, subsequently elevating the risk of pulmonary air leaks. This scenario contrasts with parenchymal lesions that cause reduced compliance, which could independently increase MAP.

This study provides accurate and detailed data from a NICU that routinely provides initial HFOV management for extremely preterm infants. However, this study had several limitations. First, this was a retrospective single-center study. Although we adjusted for potential confounders, we could not eliminate the potential influence of residual confounders. Furthermore, the lack of a difference in pulmonary air leakage between the low- and moderate-MAP groups may be due to the small number of cases. Second, multiple ventilators were used because of the long observation period (Humming V, Humming X, Humming Vue [Metran Co., Ltd., Saitama, Japan], VN500 [Drägerwerk AG & CO., Lübeck, Germany], and SLE5000 [SLE Ltd., South Croydon, UK]). We were unable to assess stroke volume or amplitude during HFOV because of ventilator differences. The effect of ventilator differences on pulmonary air leakage during the observation period is undeniable. Third, we did not have a clear protocol for setting the MAP in the HFOV or switching from HFOV to IMV. Therefore, caution should be exercised when generalizing the results of this study. Fourth, the differences in the risk of pulmonary air leakage between the MAP groups in this study may be due to differences in lung compliance or disease severity. We speculated that the lung condition itself, necessitating high MAP, may contribute to the development of pulmonary air leaks. Therefore, the findings of this study do not suggest that MAP should be managed below 12 cmH_2_O in acute-phase HFOV, but that pulmonary air leak should be a concern in the presence of respiratory diseases and general conditions that require a MAP higher than 13 cmH_2_O in acute-phase HFOV. Finally, there is no data on CO_2_ levels or radiographic findings before the pulmonary air leak occurrence. In addition, other than the somewhat subjective assessment of radiographic findings, our NICU currently lacks specific guidance on the optimal degree of lung distension that clinicians should aim for.

## Conclusions

We found that extremely preterm infants requiring acute-phase high MAP (≥13 cmH_2_O) on HFOV had a higher risk of pulmonary air leakage during the first 7 days of life. Further prospective studies are needed to determine whether high MAP (≥13 cmH_2_O) is the cause of, or merely associated with, pulmonary air leak.

## Data Availability

The raw data supporting the conclusions of this article will be made available by the authors, without undue reservation.
