# Supplementary material for: Association between mean airway pressure during high-frequency oscillatory ventilation and pulmonary air leak in extremely preterm infants during the first week of life
Source: Front Pediatr. 2024 May 30;12:1410627. doi: 10.3389/fped.2024.1410627 (PMC11171124; doi:10.3389/fped.2024.1410627)
Supplement: Supplementary file 1 [file Table1.pdf]

Supplementary Table 1. Associations between maximum MAP on acute-phase HFOV and pulmonary air leak among eligible extremely preterm infants, with HFOV rate as a potential confounder (n = 171)

	Pulmonary air leaks/ Total number	% of pulmonary air leaks	Adjusted RR	(95% CI)[a]
Maximum MAP category				
Low MAP, 7–10 cmH_2O	5/123	4.1	1	(reference)
Moderate MAP, 11–12 cmH_2O	1/27	3.7	0.9	(0.1–6.2)
High MAP, 13–15 cmH_2O	7/21	33.3	4.8	(1.4–16.9)

Data are presented as the raw number (%) and RR (95% CI).

Abbreviations: CI, confidence interval; CRIB, Clinical Risk Index for Babies; MAP, mean airway pressure; RR, risk ratio; HFOV, high-frequency oscillatory ventilation

[a]Adjusted for gestational age, pulmonary surfactant administration, prolonged premature rupture of membranes, CRIB II score, and HFOV rate[b].

[b]The HFOV rate was defined as the rate of time spent on HFOV until the onset of pulmonary air leak, seven days of age, or death before seven days of age.
